# Care-seeking patterns among families that experienced under-five child mortality in rural Rwanda

**DOI:** 10.1371/journal.pone.0190739

**Published:** 2018-01-10

**Authors:** Daniel M. Kagabo, Catherine M. Kirk, Benjamin Bakundukize, Bethany L. Hedt-Gauthier, Neil Gupta, Lisa R. Hirschhorn, Willy C. Ingabire, Dominique Rouleau, Fulgence Nkikabahizi, Catherine Mugeni, Felix Sayinzoga, Cheryl L. Amoroso

**Affiliations:** 1 Partners in Health/Inshuti Mu Buzima (PIH/IMB), Rwinkwavu, Rwanda; 2 Rwinkwavu District Hospital, Ministry of Health, Rwinkwavu, Rwanda; 3 Department of Global Health and Social Medicine, Harvard Medical School, Boston, Massachusetts, United States of America; 4 Division of Global Health Equity, Brigham and Women’s Hospital, Boston, Massachusetts, United States of America; 5 Feinberg School of Medicine, Northwestern University, Chicago, Illinois, United States of America; 6 Rwanda Biomedical Center, Ministry of Health, Kigali, Rwanda; University of South Florida, UNITED STATES

## Abstract

**Background:**

Over half of under-five deaths occur in sub-Saharan Africa and appropriate, timely, quality care is critical for saving children’s lives. This study describes the context surrounding children’s deaths from the time the illness was first noticed, through the care-seeking patterns leading up to the child’s death, and identifies factors associated with care-seeking for these children in rural Rwanda.

**Methods:**

Secondary analysis of a verbal and social autopsy study of caregivers who reported the death of a child between March 2013 to February 2014 that occurred after discharge from the child’s birth facility in southern Kayonza and Kirehe districts in Rwanda. Bivariate analyses using Fisher’s exact tests were conducted to identify child, caregiver, and household factors associated with care-seeking from the formal health system (i.e., community health worker or health facility). Factors significant at α = 0.10 significance level were considered for backwards stepwise multivariate logistic regression, stopping when remaining factors were significantly associated with care-seeking at α = 0.05 significance level.

**Results:**

Among the 516 eligible deaths among children under-five, 22.7% (n = 117) did not seek care from the health system. For those who did, the most common first point of contact was community health workers (45.8%). In multivariate logistic regression, higher maternal education (OR = 3.36, 95% CI: 1.89, 5.98), having diarrhea (OR = 4.21, 95%CI: 1.95, 9.07) or fever (OR = 2.03, 95%CI: 1.11, 3.72), full household insurance coverage (3.48, 95%CI: 1.79, 6.76), and longer duration of illness (OR = 22.19, 95%CI: 8.88, 55.48) were significantly associated with formal care-seeking.

**Conclusion:**

Interventions such as community health workers and insurance promote access to care, however a gap remains as many children had no contact with the health system prior to death and those who sought formal care still died. Further efforts are needed to respond to urgent cases in communities and further understand remaining barriers to accessing appropriate, quality care.

## Introduction

Timely and appropriate care-seeking is crucial for good health outcomes, especially among children under five years old [1]. In 2013, more than 6.3 million children died before their fifth birthday, with sub-Saharan Africa accounting for half of these deaths [2]. Integrated Management of Childhood Illness (IMCI) protocols and community case management have improved both access to care and health care workers ability to address under-five illnesses and have reduced child mortality (globally defined as any death occurring before a child’s fifth birthday) [3, 4]. However, in sub-Saharan Africa, limited access to skilled care remains among the significant factors contributing to under-five mortality [5].

Care-seeking for children is defined as actions taken by caregivers of young children in response to a child’s perceived illness. Andersen’s [6] behavioral model identifies three levels of factors that contribute to the decision to seek care or not. Predisposing factors, such as mother’s level of education [7] and the child’s gender [8] have been associated with care-seeking for children. Several enabling resources are also associated with care-seeking in low- and middle-income countries. Distance to the nearest health facility is directly proportional to risk of dying among children [9]. Financial barriers can also hinder access, with transport costs preventing or delaying care-seeking [1, 10], higher socioeconomic status associated with increased likelihood of seeking care [11, 12], and the cost of treatment being a barrier to care-seeking [13–15] that can further increase the time between presentation of symptoms and care-seeking [1]. In rural South Africa [16], nearly half of people rely on walking as the primary mode of transport to health facilities [17] and transport costs made up a large proportion of overall healthcare costs [18]. Lastly, there are a number of factors that contributed to the perceived and evaluated need to seek care, including the perception of symptoms as severe or presence of easily identifiable symptoms have been shown to prompt care-seeking for sick infants and children [7, 8, 11]. In addition, preference for seeking informal care, such as self-medication or traditional healers can also delay seeking formal care [15].

Rwanda has made tremendous progress in reducing child mortality, with one of the most rapid declines of child mortality ever recorded [19] from 152 under-five deaths per 1,000 live births in 2005 to 50 in 2015 [20]. This reduction has been attributed to a number of initiatives in the health sector, including improving geographic access by increasing the number of primary healthcare facilities [21] and reducing financial barriers through the community-based health insurance [22]. Further, specific health promotion interventions in Rwanda included insecticide-treated mosquito bed net distribution, high childhood vaccination and vitamin A supplementation coverage, implementation of community- and facility-based IMCI protocols [16], and the near-elimination of infant HIV infections [23].

Despite these gains, Rwanda has an under-five mortality rate that remains seven times higher than developed nations [20, 24]. Accessing professional healthcare plays a large role in the reduction of mortality during acute illness. However, little is known about the determinants and patterns of care-seeking prior to a child’s death in Rwanda. To continue the reduction of under-five mortality, better understanding the predictors of care-seeking behaviors among caregivers of young children in Rwanda is needed. This can inform patient-centered interventions that address factors related to delayed access to healthcare for sick children. To this end, the aim of this study is to describe the socio-demographic characteristics associated with care-seeking among families that experienced an under-five death in two rural districts in Rwanda. By focusing on care-seeking behaviors and the context around children whose illness led to the worst possible outcome of death, we hope to identify critical interventions to prevent future child mortality.

## Materials and methods

### Study design and setting

This study is a secondary analysis of data that the authors collected for a verbal and social autopsy study, which interviewed primary caregivers that experienced the death of a child under-five years of age between March 2013 to February 2014 in the Kirehe District Hospital and Rwinkwavu District Hospital catchment areas, in Kirehe and southern Kayonza Districts, respectively. In these two catchment areas, there are three levels of healthcare delivered by the Ministry of Health (MOH) with support from Partners In Health/Inshuti Mu Buzima (PIH/IMB). At the community level, there are three community health workers for each village and two of them (called “binomes”) provide services for children under-five, including the diagnosis and treatment of pneumonia, malaria, and diarrhea through community IMCI. Health centers (15 in Kirehe District and 8 in southern Kayonza District) provide mainly outpatient primary healthcare. District hospitals provide secondary care, mostly to patients referred from health centers. The most complicated cases are referred to tertiary hospitals [25], which are primarily based in the capital city of Kigali. The terrain in rural Rwanda is hilly and the average walking distance from households to the nearest health facility in Kirehe is 92.4 minutes and 64.2 minutes in Kayonza [26]. The most common forms of transportation in these districts are walking and fee-per-use methods such as bicycles, minibuses, and motorcycle taxis. The STROBE checklist (S1 File) is available in the supplementary materials.

### Participants and data collection

The original data collection aimed to have a census of all under-five deaths during the study period. For the main verbal and social autopsy study, all under-five deaths during the study period were identified from community and health facility records by triangulating existing MOH reporting systems and the Monitoring of Vital Events using Information Technology (MoVe-IT) program. MoVe-IT was introduced in the two districts in 2012 to complement existing reporting systems, and consisted of text message reporting by community health workers of all vital events among mothers and under-five children [27]. Families that experienced an under-five death were located in the community with the help of community health workers. For this secondary analysis, all children under-five that died and were captured in the original data collection were included unless they met the exclusion criteria for our secondary analysis: neonatal deaths that occurred before discharge from the facility where they were born and children whose caregivers did not report on care-seeking were excluded. Those born outside of a facility were included if the caregivers reported on care-seeking.

**Fig 1:**
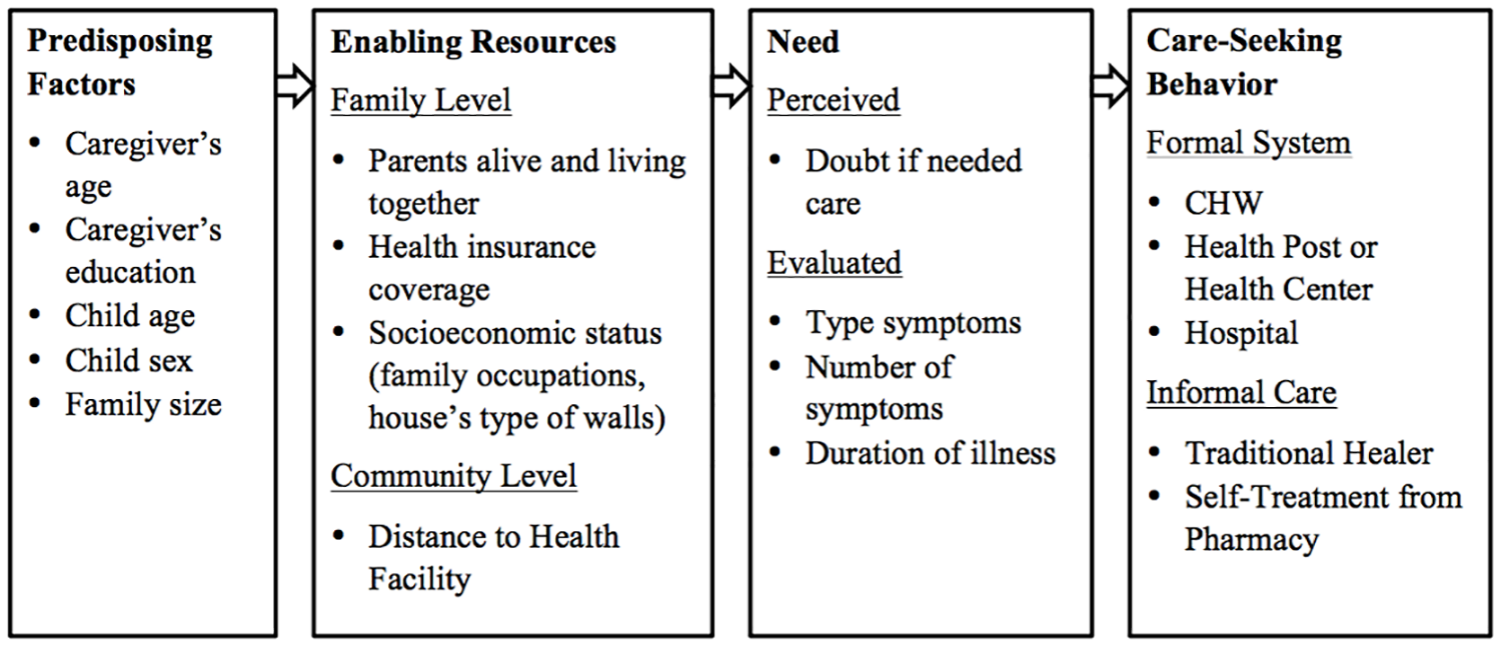
Behavioral model of predictors of care-seeking behavior.

Data was collected through household interviews with caregivers of the deceased child. Caregivers were asked to report on demographic characteristics, and the symptoms and care-seeking patterns prior to the child’s death using a structured interview questionnaire adapted from the World Health Organization’s (WHO) verbal autopsy tool [28]. The WHO’s verbal autopsy tool contains a series of questions about specific symptoms the child experienced during the illness that resulted in the child’s death, such as presence of a fever or diarrhea, the duration of the illness prior to death, as well as contextual factors such as whether the child was involved in an accident, distance of the household to a health facility, and caregiver perception of illness severity. The questionnaire also included a subset of questions from the MOH’s Death Audit Tool (i.e, place of birth, places where care was sought, reasons for not seeking care, time spent in health facilities, perceived quality of care, status of mother and father, household religion, household occupation, and health insurance coverage) and the 2010 Rwanda Demographic and Health Survey (i.e., household assets and housing materials). Variables were grouped into predisposing factors, enabling factors, and perceived and evaluated need based on the Andersen framework for care-seeking (Fig 1).[6]

No personal identifiers were stored on the data collection form; instead, caregivers’ identifiers were stored in a separate file in a secure location with matching study IDs for data cleaning and validation. Initial data were collected on paper with double entry for data quality control but the majority of data were collected using Android tablets.

### Analysis

The primary outcome of interest in this study was formal care-seeking prior to the child’s death, which was defined as seeking care from the formal health system through community health workers or health facilities (health posts, health centers, hospitals, or private clinics). Taking a child to traditional healers or seeking self-treatment from a pharmacy or from neighbors were all categorized as not seeking formal care.

We describe child, caregiver and household variables and test for their associations with care-seeking using a Fisher’s exact test. All variables associated with care-seeking at α = 0.10 significance level in the bivariate analyses were entered into a full logistic regression model. We used backwards stepwise regression to identify predictors and risk factors associated with seeking care upon noticing the child’s illness among children under-five, stopping when all remaining variables were significant at the α = 0.05 significance level. All analyses were completed in Stata version 13 (StataCorp 2011. College Station, TX).

### Ethical approval

This study was approved by Rwanda National Ethics Committee (RNEC) and the Partners Institutional Review Board in Boston, Massachusetts under the Population Health Implementation and Training (PHIT) program, a partnership between PIH/IMB, the University of Rwanda and the Rwanda MOH. All caregivers who participated provided written informed consent. Given the sensitive nature of the questionnaire, all data collectors were trained to be considerate of and sensitive to the caregiver’s needs such as giving time to respond, ensuring caregivers understood that all answers were voluntary, providing comfort in any instance where a caregiver may have become upset, and postponing or ending the interview if preferred by the caregivers.

## Results

**Fig 2:**
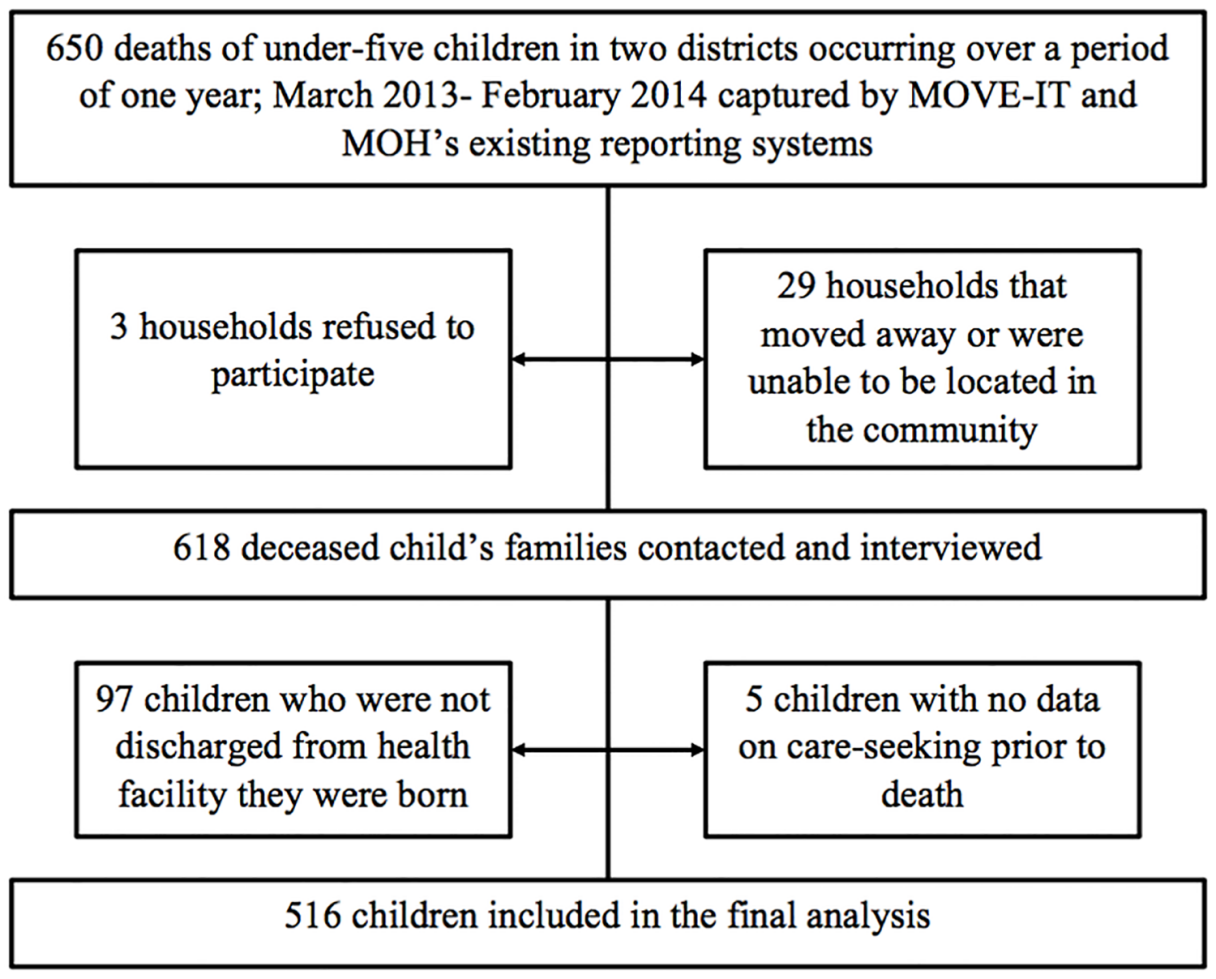
Study participants.

In the original verbal and social autopsy study, 618 out of 650 households with an under five death between March 2013 to February 2014 were visited; 5% of families were not interviewed due to caregiver refusal, relocation or being unable to be found based on the information contained in facility, community, and MoVe-IT death records (n = 29, see Fig 2). Of the 618 under-five children whose caregivers were interviewed, 97 died before discharge from the health facility where they were born, leaving 521 deaths available for this study. Of these, the respondent was unsure whether care was sought for five children (1.0%) and these children were excluded, leaving 516 in the final sample.

Half of children who died were above one year of age (50.2%; n = 259), while 35.1% (n = 181) were infants aged 28 days to 1 year, and 14.7% (n = 76) were neonates (Table 1). Fifty-five percent of the children were male (54.5%, n = 281). The most common symptoms for children in our sample were fever (63.3%, n = 322), breathing problems (55.2%, n = 284), vomiting (45.0%, n = 232) and diarrhea (32.8%, n = 168). Less common symptoms were convulsions (20.6%, n = 105/510) or unconsciousness (16.2%, n = 77/475). A small number (4.3%, n = 22) experienced an accident or injury that led to their death. For nearly all children (97.5%, n = 499/512), both parents were still alive at the time of death. Duration of the child’s illness prior to death varied with 28.9% (n = 149) of the children ill for over one week, 32.6% (n = 168) ill between three to seven days, 21.1%, (n = 109) ill for 12 hours to two days, and 17.4% (n = 90) were ill for less than 12 hours.

**Table 1:** Descriptive characteristics of the child, caregivers, and household.

	N = 516	
	n	%
**Child Demographic and Illness Characteristics**		
Age		
Neonatal (0–28 days)	76	14.7
Infant (29 days—11 months)	181	35.1
Child (12–59 months)	259	50.2
Sex		
Male	281	54.5
Female	235	45.5
Symptoms		
*Fever (n = 509)*		
No	187	36.7
Yes	322	63.3
*Breathing problem (n = 515)*		
No	231	44.9
Yes	284	55.2
*Diarrhea (n = 516)*		
No	344	67.2
Yes	168	32.8
*Vomit (n = 516)*		
No	284	55.0
Yes	232	45.0
*Unconscious (n = 475)*		
No	398	83.8
Yes	77	16.2
*Convulsions (n = 510)*		
No	405	79.4
Yes	105	20.6
*Number of symptoms (n = 516)*		
No symptom	57	11.1
1 symptom	84	16.3
2 symptoms	101	19.6
3+ symptoms	274	53.1
Child was involved in an injury or accident (n = 516)		
No	494	95.7
Yes	22	4.3
Duration of illness (n = 516)		
Less than 12 hours	90	17.4
12 to 48 hours	109	21.1
3 to 7 days	168	32.6
Greater than 1 week	149	28.9
**Caregiver Demographics**		
Mother and Father alive (n = 512)		
No	13	2.5
Yes	499	97.5
Parents living together (n = 499)		
No	115	23.1
Yes	384	77.0
Mother’s occupation (n = 511)		
Non- agricultural sector	27	5.3
Agricultural sector	484	94.7
Mother’s age (years) (n = 492)		
<20	14	2.9
20–29	207	42.1
30–39	203	41.3
40–49	66	13.4
50+	2	0.4
Mother’s education level (n = 491)		
No formal education	150	30.6
Primary or higher	341	69.5
Father’s occupation (n = 500)		
Non- agricultural sector	67	13.4
Agricultural sector	433	86.6
Father’s age (years) (n = 384)		
20–29	111	28.9
30–39	149	38.8
40–49	85	22.1
50+	39	10.2
Father’s education (n = 389)		
No formal education	103	26.5
Primary or higher	286	73.5
**Household Characteristics**		
Household size (n = 512)		
1–3 living members	195	38.1
4–6 living members	253	49.4
7+ living members	64	12.5
Number of dependents aged 15 or younger (n = 516)		
No other child dependents	24	4.7
1–2 other dependents	225	43.6
3–4 other dependents	165	32.0
5+ other dependents	102	19.8
Types of house exterior walls (n = 516)		
Unfinished walls	453	87.8
Finished walls	63	12.2
Availability of electricity (n = 515)		
No	478	92.8
Yes	37	7.2
Household has a mosquito net (n = 515)		
No	45	8.7
Yes	470	91.3
Household insurance coverage for living members (n = 506)		
No Insurance	104	20.5
Some members insured	68	13.4
All members insured	334	66.0
Travel time to nearest health facility is over 2 hours walking distance (n = 514)		
No	321	62.5
Yes	193	37.6
Caregiver’s perceived quality of care at the nearest health facility (n = 504)		
Very good	23	4.6
Good	335	66.5
Moderate	87	17.3
Poor	59	11.7
Doubted if child needed medical care (n = 513)		
No	346	67.5
Yes	167	32.6
Used traditional medicine during the child’s final illness (n = 516)		
No	381	73.8
Yes	135	26.2
Challenges experienced by the household when seeking healthcare (n = 516)		
*Getting money to pay for treatment*		
No	219	42.4
Yes	297	57.6
*Distance from nearest health facility*		
No	301	58.3
Yes	215	41.7
*Not wanting to go alone*		
No	403	78.1
Yes	113	21.9
*Getting permission to see the doctor*		
No	492	95.4
Yes	24	4.7

The majority of children’s parents were living together (77.0%, n = 384/499) and 94.7% of mothers (n = 484/511) and 86.6% of fathers (n = 433/500) worked in agriculture. For the 492 mothers with age recorded, most were aged 20–29 (42.1%, n = 207) or 30–39 (41.3%, n = 203). For the 384 fathers with age recorded, 28.9% (n = 111) were 20–29, 38.8% (n = 149) were age 30–39, 22.1% (n = 85) were age 40–49 and 10.2% (n = 39) were over age 50. A third of mothers (30.6%, n = 150/491) and a quarter of fathers (26.5%, n = 103/389) reported having no formal education. The majority of families (87.8%, n = 453) had unfinished exterior walls (walls that were not cemented) and lacked access to electricity in their households (92.8%, n = 478/515). Two-thirds of families (66.0%, n = 334/506) had insurance for all family members, and 20.5% had no insured family members (n = 104/506). Over one-third of households (37.6%, n = 193/514) reported living more than two hours walking distance from the nearest health facility. When asked what factors pose problems for the household when seeking healthcare in general, the most common responses were getting money to pay for treatment (57.6%, n = 297) and distance to the nearest health facility (41.7%, n = 215).

Overall, in 22.7% (n = 117) of the under-five deaths, the caregiver did not seek any formal healthcare prior to the child’s death (Table 2). Of caregivers who sought formal care prior to the child’s death, 45.8% (n = 182) reported that the first point of care was community health workers and 42.5% (n = 168) reported going first to a health center or other outpatient clinic.

**Table 2:** Formal and informal care seeking prior to the child’s death.

	Total		Sought formal care		No care	
	N = 516		N = 399 (77.3%)		N = 117 (22.7%)	
	n	%	n	%	n	%
Places visited at any point during child’s illness						
Traditional healer	139	27.0	119	29.8	20	17.1
Pharmacy	21	4.1	18	4.5	3	2.6
Community health worker	233	45.2	233	58.4	-	-
Health post, health center, or other clinic	335	64.9	335	84.0	-	-
District or referral hospital	134	26.0	134	33.6	-	-
First place visited by the caregiver	N = 416		N = 395		N = 21	
Traditional healer	50	12.0	31	7.9	19	90.4
Pharmacy	5	1.2	3	1.0	2	9.5
Community health worker	182	43.3	182	45.8	-	-
Health post, health center, private clinic	168	40.0	168	42.5	-	-
District or referral hospital	11	2.6	11	2.8	-	-

Thirty-four (8.9%) caregivers who eventually sought formal care first pursued self-treatment from a traditional healer (7.9%, n = 31) or pharmacy (1.0%, n = 3). Over half of caregivers that sought formal care went to a community health worker at some point during the illness (58.4%, n = 233), nearly all visited a health center or other outpatient clinic (84.0%, n = 335), and a third went to a hospital (33.6%, n = 134). Of caregivers that did not seek any formal care, 19.7% (n = 23/117) visited a traditional healer or sought self-treatment from a pharmacy. Caregivers who did not seek formal care prior to the child’s death were asked to report reasons for not seeking care upon noticing the child’s illness. The most commonly reported reason for not seeking formal care was the perception that the child fell ill and died quickly (45.3%, n = 53). Only 17.1% (n = 20) of the caregivers reported finances being a barrier and 18.0% (n = 21) reported not thinking the illness was serious.

Care-seeking patterns were significantly different depending on the child’s age (p = 0.04, Table 3). Children with fever (p<0.001), breathing problems (p<0.001), diarrhea (p<0.001), vomiting (p<0.001) and unconsciousness (p = 0.023) were more likely to have sought formal care. Children with three or more symptoms nearly always had formal care (90.9%, n = 249/274) compared to 38.6% (n = 22/274) of children who did not exhibit one of the six most common symptoms (p<0.001). Significantly more children involved in an accident or injury that led to their death received no formal care (54.5%, n = 12/22) compared to those not involved in an accident or injury (21.3%, n = 105/494, p = 0.001). Children with symptoms lasting less than 12 hours were less likely to have formal care (64.4%, n = 58/90) compared to those with symptoms lasting over a week (6.7%, n = 10/149, p<0.001). A third of mothers with no education did not seek formal care for their children (35.3%, n = 53/150) compared to 17.3% (n = 59/341) among those with primary education or higher (p<0.001). A higher portion of young fathers aged 20–29 sought formal care (85.6%, n = 95/111) compared older fathers (p = 0.036). Households with all members insured were significantly more likely to seek care with 82.9% (n = 277/334) as compared to 66.3% (n = 69/104) of those with no insurance sought care (p<0.001). Households that were over two hours from the nearest health facility were less likely to seek formal care (27.5%, n = 53/193) as compared to those who lived closer (19.6%, n = 63/231, p = 0.050). Self-reported barriers to care such as getting money to pay, distance, not wanting to go alone, and getting permission were not associated with ultimately seeking care prior to child’s death.

**Table 3:** Bivariate associations between care-seeking and child, caregiver, and household characteristics.

	Sought formal care		No care		P-value
	N = 399 (77.3%)		N = 117 (22.7%)		
	n	%	n	%	
**Child Demographic and Illness Characteristics**					
Age					
Neonatal (0–28 days)	50	65.8	26	34.2	0.037
Infant (29 days—11 months)	142	78.5	39	21.5	
Child (12–59 months)	207	79.9	52	20.1	
Sex					
Male	216	76.9	65	23.1	0.833
Female	183	77.9	52	22.1	
Symptoms					
*Fever (n = 509)*					
No	112	59.9	75	40.1	<0.001
Yes	281	87.3	41	12.7	
*Breathing problem (n = 515)*					
No	156	67.5	75	32.5	<0.001
Yes	243	85.6	41	14.4	
*Diarrhea (n = 516)*					
No	240	69.8	104	30.2	<0.001
Yes	155	92.3	13	7.7	
*Vomit (n = 516)*					
No	198	69.7	86	30.3	<0.001
Yes	201	86.6	31	13.4	
*Unconscious (n = 475)*					
No	304	76.4	94	23.6	0.023
Yes	68	88.3	9	11.7	
*Convulsions (n = 510)*					
No	310	76.5	95	23.5	0.293
Yes	86	81.9	19	18.1	
*Number of symptoms (n = 516)*					
No symptom	22	38.6	35	61.4	<0.001
1 symptom	52	61.9	32	38.1	
2 symptoms	76	75.2	25	24.8	
3+ symptoms	249	90.9	25	9.1	
Child was involved in an injury or accident (n = 516)					
No	389	78.7	105	21.3	0.001
Yes	10	45.5	12	54.5	
Duration of illness (n = 516)					
Less than 12 hours	32	35.6	58	64.4	<0.001
12 to 48 hours	80	73.4	29	26.6	
3 to 7 days	148	88.1	20	11.9	
Greater than 1 week	139	93.3	10	6.7	
**Caregiver Demographics**					
Mother and Father alive (n = 512)					
No	11	84.6	2	15.4	0.742
Yes	385	77.2	114	22.8	
Parents living together (n = 499)					
No	89	77.4	26	22.6	1.000
Yes	296	77.1	88	22.9	
Mother’s occupation (n = 511)					
Non- agricultural sector	22	81.5	5	18.5	0.813
Agricultural sector	374	77.3	110	22.7	
Mother’s age (years) (n = 492)					
<20	12	85.7	2	14.3	0.086
20–29	170	82.1	37	17.9	
30–39	146	71.9	57	28.1	
40–49	51	77.3	15	22.7	
50+	1	50.0	1	50.0	
Mother’s education level (n = 491)					
No formal education	97	64.7	53	35.3	<0.001
Primary or higher	282	82.7	59	17.3	
Father’s occupation (n = 500)					
Non- agricultural sector	51	76.1	16	23.9	0.876
Agricultural sector	334	77.1	99	22.9	
Father’s age (years) (n = 384)					
20–29	95	85.6	16	14.4	0.036
30–39	106	71.1	43	28.9	
40–49	67	78.8	18	21.2	
50+	28	71.8	11	28.2	
Father’s education (n = 389)					
No formal education	83	80.6	20	19.4	0.341
Primary or higher	216	75.5	70	24.5	
**Household Characteristics**					
Household size (n = 512)					
1–3 living members	156	80.0	39	20.0	0.362
4–6 living members	193	76.3	60	23.7	
7+ living members	46	71.9	18	28.1	
Number of dependents aged 15 or younger (n = 516)					
No other child dependents	18	75.0	6	25.0	0.421
1–2 other dependents	172	76.4	53	23.6	
3–4 other dependents	124	75.2	41	24.8	
5+ other dependents	85	83.3	17	16.7	
Types of house exterior walls (n = 516)					
Unfinished walls	351	77.5	102	22.5	0.872
Finished walls	48	76.2	15	23.8	
Availability of electricity (n = 515)					
No	372	77.8	106	22.2	0.540
Yes	27	73.0	10	27.0	
Household has a mosquito net (n = 515)					
No	32	71.1	13	28.9	0.351
Yes	336	76.4	104	23.6	
Household insurance coverage for living members (n = 506)					
No Insurance	69	66.3	35	33.7	<0.001
Some members insured	45	66.2	23	33.8	
All members insured	277	82.9	57	17.1	
Travel time to nearest health facility is over 2 hours walking distance (n = 514)					
No	258	80.4	63	19.6	0.050
Yes	140	72.5	53	27.5	
Caregiver’s perceived quality of care at the nearest health facility (n = 504)					
Very good	20	87.0	3	13.0	0.567
Good	257	76.7	78	23.3	
Moderate	69	79.3	18	20.7	
Poor	49	83.1	10	16.9	
Doubted if child needed medical care (n = 513)					
No	269	77.7	77	22.3	0.822
Yes	128	76.6	39	23.4	
Used traditional medicine during the child’s final illness (n = 516)					
No	290	76.1	91	23.9	0.285
Yes	109	80.7	26	19.3	
Challenges experienced by the household when seeking healthcare (n = 516)					
*Getting money to pay for treatment*					
No	176	80.4	43	19.6	0.168
Yes	223	75.1	74	24.9	
*Distance from nearest health facility*					
No	240	79.7	61	20.3	0.136
Yes	159	74.0	56	26.0	
*Not wanting to go alone*					
No	316	78.4	87	21.6	0.309
Yes	83	73.5	30	26.5	
*Getting permission to see the doctor*					
No	381	77.4	111	22.6	0.803
Yes	18	75.0	6	25.0	

In the final multivariate model (Table 4), caregivers were more likely to seek care if the child presented with a fever (Odds Ratio [OR] = 2.03, 95% Confidence Interval [CI]: 1.11, 3.72, p = 0.022), diarrhea (OR = 4.21, 95% CI: 1.95, 9.07, p<0.001), had an illness duration of greater than 12 hours (ill from 12 hours to 48 hours, OR = 4.51, 95% CI: 2.16, 9.42, p<0.001; from three to seven days, OR = 13.70, 95% CI: 6.04, 31.07, p<0.001; and over one week, OR = 22.19, 95% CI: 8.88, 55.48, p<0.001 compared to illness duration less than 12 hours), had a higher level of maternal education (OR = 3.36, 95% CI: 1.89, 5.98, p<0.001), and if the household was fully insured (OR = 3.48, 95% CI: 1.79, 6.76, p<0.001).

**Table 4:** Multivariate logistic regression of care-seeking predictors.

Predictor	Full Model				Final Model			
	95% CI				95% CI			
	OR	Lower	Upper	P-value	OR	Lower	Upper	P-value
Child’s age at death								
Neonate <28 days	ref							
Infant 1–11 months	1.06	0.35	3.23	0.923				
Child 12–59 months	0.88	0.30	2.58	0.811				
Child had a fever								
No	ref							
Yes	2.12	0.77	5.83	0.145	2.03	1.11	3.72	0.022
Child had breathing problems								
No	ref							
Yes	1.32	0.49	3.54	0.586				
Child had diarrhea								
No	ref							
Yes	10.72	2.81	40.86	0.001	4.21	1.95	9.07	<0.001
Child was vomiting								
No	ref							
Yes	0.79	0.29	2.16	0.646				
Child was unconscious								
No	ref							
Yes	2.30	0.62	8.57	0.213				
Number of symptoms								
No symptoms	ref							
1 symptoms	0.51	0.13	1.95	0.326				
2 symptoms	0.76	0.14	4.21	0.754				
3+ symptoms	1.02	0.12	8.41	0.987				
Child was involved in an injury or accident								
No	ref							
Yes	0.95	0.17	5.32	0.958				
Duration of child’s illness								
<12 hours	ref							
12-48hrs	7.27	2.46	21.54	<0.001	4.51	2.16	9.42	<0.001
3-7days	29.36	8.92	96.72	<0.001	13.70	6.04	31.07	<0.001
Over 1 week	41.93	11.07	158.74	<0.001	22.19	8.88	55.48	<0.001
Mother’s level of education								
No formal education	ref							
Primary education or higher	3.84	1.74	8.46	0.001	3.36	1.89	5.98	<0.001
Father’s age								
20–29	ref							
30–39	0.48	0.18	1.30	0.148				
40–49	0.69	0.22	2.13	0.523				
50+	0.36	0.10	1.35	0.129				
Family insurance coverage								
None insured	ref							
Some Insured	2.14	0.57	8.09	0.261	1.42	0.59	3.43	0.440
All Insured	4.73	1.90	11.78	0.001	3.48	1.79	6.76	<0.001
Travel time to nearest health facility is over 2 hours walking distance								
No	ref							
Yes	0.51	0.24	1.10	0.086				

## Discussion

In our study, we found that nearly a quarter of children under-five that died in Kirehe and southern Kayonza Districts during the study period had no care during their final illness. While the majority of children did access the formal health system, the care received was not able to prevent the child’s death. For those who sought formal care, Rwanda’s large network of community health workers was an important resource and the most common first point of care. Factors at all levels of Andersen’s care-seeking model predicted whether a child ultimately had contact with the formal health care system prior to death—the predisposing factor of maternal education, the enabling factor of household health insurance coverage, and several factors related to the severity of the child’s illness and symptoms [6]. It is vital to understand care-seeking behavior prior to death in order to identify facilitating factors and barriers to accessing timely and appropriate care in rural parts of Rwanda and develop interventions to prevent unnecessary child deaths in the future.

In our study, longer duration of illness was a strong predictor of care-seeking; for caregivers who did not seek care, the caregiver reporting the death occurred quickly was the main reason provided for not seeking care. There are several factors identified in other studies that could contribute to this finding including lack of awareness of the severity of the child’s illness [29], first attempting traditional medicines or self-treatment [30], as well as lack of access to timely transportation when an illness occurs quickly. A number of barriers may create challenges for accessing care when a death occurs quickly, such as incidents of injuries, drowning, or other accidents. This may also be associated with caregivers’ preferring to seek care during the day due to safety concerns at night [31] or limited accessibility of nighttime travel. Additionally, limited access to emergency transportation poses challenges for quick, affordable access to care [32]. In Rwanda, ambulance use is limited for responding to community emergencies; ambulances are primarily used for inter-health facilities transfers or traffic accidents [33]. Interventions which might address these challenges could include having the one male community health worker in the “binome” pair accompany caregivers for night time emergencies to help ensure safety of the child and caregiver while also increasing access to care during this critical period. Further, strengthening the emergency transport system to facilitate urgent transfers from the community to a health facility is needed.

We also found that the symptoms related to the child’s illness were important factors for whether or not formal care was sought. Similar to a study in Tanzania [34], we found that caregivers were significantly more likely to seek care if a child had fever or diarrhea. This may be due in part to the contribution of community health workers who, under the national community health program, have sensitized parents about danger signs and the importance of seeking care whenever children present with these noticeable symptoms [30]. Further, community health workers are trained in IMCI to detect, treat and provide referrals as appropriate for common childhood illnesses [16]. It is noted however that signs of respiratory distress were not associated with care-seeking, and further investigation is required to determine if the messaging to families about when to seek care when these symptoms are present can be improved. In addition to helping with the early identification of common symptoms, community health workers need to emphasize the importance of timely care-seeking by caregivers before their children’s health becomes critical.

While household wealth was not directly measured, we believe that the majority of households in this study were likely to be of lower socioeconomic status, with limited access to electricity and homes primarily constructed with unfinished walls. These findings indicate slightly lower socioeconomic standing of the households in our study than a national, population survey which found in the Eastern Province that 15.3% of households have electricity and 63.4% of individuals were living in houses with unfinished walls [35]. However, and perhaps surprisingly given that associated costs are often a barrier for care-seeking [36], none of these indicators of poor socioeconomic status were associated with care-seeking in our results and for those who did not seek care, very few indicated limited finances as a barrier. This may be explained by the focus of Rwanda’s health policies on reducing social inequity [37] or that there is limited variability in socioeconomic status in this study population. Community-based health insurance, called *mutuelle de santé*, has increased care-seeking by reducing financial barriers to care, with enrolled families having twice the healthcare utilization rates compared to uninsured families [38]. In 2010, household enrollment in *mutuelle de santé* peaked at 90% [14], and later decreased to 73% household enrollment in 2014 [39]. Rwanda’s community-based health insurance system may increase access to care by preventing catastrophic out of the pocket payment whenever family members are sick [22], and this insurance is subsidized with co-payment exemptions for the poorest households [40]. The possibility that subsidized insurance for the poorest households reduces the impact of poverty on care-seeking behavior is also supported by the finding that households with all family members were covered by health insurance were significantly more likely to take the child for care when ill. Additionally, cost barriers may be difficult for families to discuss, given that the ultimate outcome was the child’s death.

Limitations of this study should be noted. Information about what occurred during the time leading up to the child’s death was based on caregiver’s recall of specific symptoms and health care-seeking patterns. However, we aimed to minimize recall bias by interviewing caregivers within twelve months after the child’s death. Further, there was the potential for desirability bias, where individuals may not be fully forthcoming about their child’s death, wanting to shield themselves from blame. However, only assessing care-seeking behaviors for children who died should minimize the bias as we are not comparing them to children who survived a similar illness. Finally, it is noted that this study only includes care-seeking behaviors among families that experienced an under-five death. While this may not be generalizable to all families with children under-five, it is still important because its sheds light on scarcely researched care-seeking patterns in rural Rwanda and challenges faced by caregivers in critical time of an under-five child.

## Conclusions

As we reflect on the commendable achievement in improved health in Rwanda, these findings highlight that barriers remain to timely care-seeking for critically ill children in rural Rwanda with nearly a quarter of children who died having no contact with the formal health system prior to death. With the majority of the deceased children having accessed the formal health system, further research should explore the care experiences and quality of care services received. Efforts to decentralize primary health care, and to promote integrated community case management and health insurance have contributed to reducing child mortality, but more must be done to maintain this momentum and ensure all children have access to potentially lifesaving services. Community health workers remain a vital first point-of-care for families and in creating awareness of childhood illness danger signs and also in encouraging timely care-seeking. Another significant point-of-care is primary health centers. Nevertheless, this study highlights important challenges with responding to urgent cases in rural communities and further assessment is needed to determine feasibility and efficiency of enhanced expanded emergency response systems and improve the quality of care to save lives.

## Supporting information

S1 File STROBE checklist. (DOC)
